# Drop homotopic effects of masseter-muscle pain on somatosensory sensitivity in healthy participants

**DOI:** 10.1038/s41598-021-89937-3

**Published:** 2021-05-19

**Authors:** Hidetoshi Hayakawa, Takashi Iida, Mika Honda-Sakaki, Manabu Masuda, Peter Svensson, Osamu Komiyama

**Affiliations:** 1 Division of Oral Function and Rehabilitation, Department of Oral Health Science, Nihon University School of Dentistry at Matsudo, 2-870-1, Sakaecho-nishi, Matsudo, Chiba 271-8587 Japan; 2 Section of Orofacial Pain and Jaw Function, Department of Dentistry and Oral Health, Aarhus University, Aarhus, Denmark; 3 Scandinavian Center for Orofacial Neurosciences (SCON), Aarhus, Denmark; 4 Department of Orofacial Pain and Jaw Function, Faculty of Odontology, Malmø University, Malmö, Sweden

**Keywords:** Touch receptors, Neuroscience, Somatosensory system, Pain, Chronic pain

## Abstract

Current pain classifications use 1.0-kg palpation of the masseter muscle to distinguish between “pain patients” and “healthy controls” but a thorough understanding of the normal physiological responses to various somatosensory stimuli is lacking. The aim of this study was to investigate somatosensory function of the skin over the masseter muscle in healthy participants that were divided into a masseter pain prone group (MPP) (n = 22) and non-MPP group (n = 22), according to the response to a 1.0-kg palpation. Quantitative sensory testing (QST) was performed at the skin above the right masseter muscle (homotopic). In an additional experiment, 13 individuals each from MPP and non-MPP received application of 60% topical lidocaine tape to the skin over the masseter muscle for 30 min. Immediately after, mechanical pain sensitivity (MPS), dynamic mechanical allodynia, and pressure pain threshold were tested. Homotopic MPS was significantly higher and PPTs significantly lower in MPP than in N-MPP (P < 0.05). Strikingly, no other differences in QST outcomes were observed between the groups (P > 0.05). After lidocaine application, no significant differences in homotopic MPS were observed between groups. The presence or absence of acute provoked pain in masseter muscle is exclusively associated with differences in homotopic MPS which is decreased following topical anesthesia.

## Introduction

Current international classifications of myofascial orofacial pain rely heavily on the response to palpation of the jaw muscles^1–3^. To distinguish between “pain” and “healthy” an arbitrary palpation pressure of 1.0 kg for 2 s has been recommended, however, it is a common clinical observation that also otherwise healthy and pain-free individuals may report pain on such standardized palpation with 1.0 kg^4^. It is not known if such a difference in responsiveness which could be conceptualized as being “masseter pain prone” (MPP) has any bearing on other somatosensory stimuli and effect of topical anesthesia within the same region (homotopic site). The present study aimed to explore the normal somatosensory physiology in order better to comprehend the pathophysiology involved in chronic orofacial pain.

The pathophysiology of myofascial orofacial pain is, indeed, not well understood and has recently been suggested to be termed “primary” pain, i.e., a specific cause for the pain cannot be identified and pain is no longer a symptom but rather a disease or disorder in its own right^3,5^. defined as pain in the masticatory muscles, with or without functional impairment, and not attributable to any other disorder^3^. A previous study has demonstrated that experimental masseter muscle pain induced by glutamate injections influence either pain intensity or pressure pain sensitivity in the masseter muscle^6,7^, moreover other studies have indicated a significant sensitization of the homotopic muscle following noxious stimulation with glutamate injections^8–10^. Costa et al. reported that short-lasting experimental muscle pain was capable of causing loss of tactile sensitivity and perceptual distortions of the face^11^. It has also been shown that longer-lasting pain in the masseter muscle caused by continuous infusion of hypertonic saline is associated with significantly higher sensitivity over the skin of the masseter muscle^7^. The significance of being MMP to a brief (2 s) 1.0 kg palpation stimulation is therefore not clear.

The German Research Network on Neuropathic Pain (DFNS) has recommended a protocol of 13 quantitative sensory testing (QST) measures for detecting somatosensory abnormalities^12^. Pigg et al. evaluated the inter- and intra-examiner reliabilities of QST measures for assessing somatosensory function and concluded that the reliability of QST in the orofacial area is adequate for future application of the method, such as for the establishment of normative values^13^. Moreover, Costa et al. investigated short-lasting experimental muscle pain by applying QST measures to the skin over the masseter muscle, and suggested a capacity for causing loss of tactile sensitivity as well as perceptual distortion of the face regardless of preconditioning with a topical lidocaine patch. In addition, short-term application of a lidocaine patch did not significantly affect the mechanical somatosensory profile^14^. Assessment of the effect of pressure-evoked masseter muscle pain on somatosensory sensitivity with topical lidocaine patches may thus be useful for obtaining a deeper understanding of somatosensory sensitivity of the skin overlying the masseter muscle (homotopic site) in both MPP and non-MPP individuals. However, to date, no studies have investigated changes in somatosensory sensitivity following topical lidocaine patches in MPP and non-MPP individuals.

The specific hypothesis in the present study was that significant differences in homotopic somatosensory function would be detected between healthy participants with and without pressure-evoked masseter muscle pain. The main aim of this study was to investigate the normal physiological mechanisms associated with acute masseter muscle pain in order better to understand somatosensory abnormalities in patients with chronic masseter muscle pain.

## Materials and methods

### Participants

Forty-four participants (22 men, mean ± standard deviation [SD] age, 27.3 ± 3.2 years; 22 women, mean age 27.6 ± 2.6 years) were recruited from the community of students and staff members at Nihon University, Chiba, Japan. Inclusion criteria were as follows: age > 18 years; unassisted pain-free jaw opening, > 40 mm; and no pain during maximum unassisted or assisted jaw-opening movements. The number of participants were calculated by power analysis^15^. Exclusion criteria comprised: pregnancy; any mental disorder; allergy to lidocaine; scheduled dental treatment as of the time of the study; or intake of medications (analgesics, antidepressants, or hypnotics) within 48 h of the investigation^16^. The Patient Health Questionnaire (PHQ-9, PHQ-15), Generalized Anxiety Disorder (GAD-7) were used to screen for depression, somatic symptoms, and anxiety disorder severity. The Score of PHQ-9, PHQ-15, GAD-7 in all participants were within normal range. Prior to enrollment in the study, all participants received written and oral explanations about the experiment and provided their informed written consent to participate. The present study was conducted in accordance with the guidelines established by the Declaration of Helsinki, and all protocols were approved by the ethics committee of Nihon University School of Dentistry at Matsudo (EC 18-024).

### Study design

**Figure 1:**
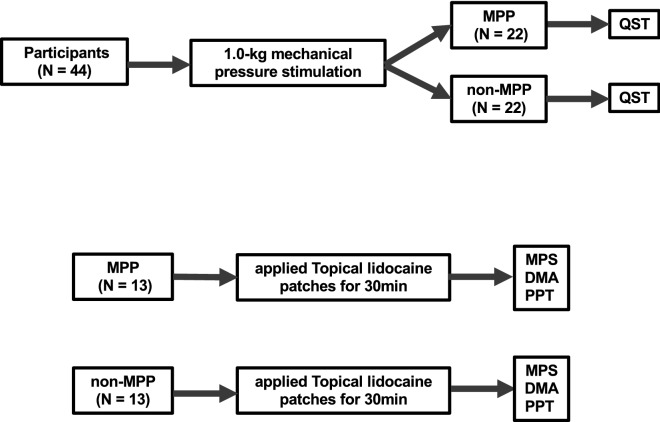
Flowchart of the main experiment procedure and the additional experiment procedure.

The present study comprised two experiments, as a main experiment and an additional experiment. During the experiment, participants were seated on a comfortable chair in a relaxed state. First, 44 participants were divided into a masseter muscle pain prone (MPP) group (n = 22) and a non-masseter muscle pain prone (non-MPP) group (n = 22), according to the response to a 1.0-kg mechanical pressure stimulation for 2 s to the center of the right masseter muscle, using a mechanical device (PALPETER; Sunstar Swiss SA, Swiss, 1.0 kg) to standardize the site and force of the palpation (Fig. 1)^17^. The center of the right masseter muscle was identified by palpation during repetitive clenching. After application of the pressure stimulus, participants were asked to answer the presence/absence of pain during palpation.

In the main experiment, all 22 individuals of the MPP group (9 men, 13 women), and 22 individuals of the non-MPP group (13 men, 9 women) participated. QST was performed on the right masseter, the skin over the center of the right masseter, and the right first dorsal interosseous (FDI) muscle as a control. QST was conducted according to the methods proposed by the DFNS (Fig. 1).

In the additional experiment, 13 individuals from the MPP group (5 men, 8 women) and 13 individuals from the non-MPP group (8 men, 5 women) participated. In this experiment, 1 cm^2^ of 60% topical lidocaine tape (PENLES TAPE; Maruho, Osaka, Japan) was applied to the skin over the center of the right masseter and right FDI for 30 min^18^. After application, mechanical pain sensitivity (MPS), dynamic mechanical allodynia (DMA), and pressure pain threshold (PPT) were performed at the right masseter, the skin over the center of the right masseter, and the right FDI (Fig. 1).

In the MPP group, QST was assessed at the point where participants reported masseter pain during palpation with the standardized 1.0 kg pressure. In the non-MPP group, the center of the masseter muscle was used as test site. Moreover, for the heterotopic site, the central part of the FDI was tested.

### Quantitative sensory testing (QST)

The standardized battery for QST applied to the right masseter muscle and right FDI involved 13 thermal and mechanical tests^13,19^. In this study, QST was performed using the following method for both the main and additional experiments. These tests included cold detection threshold (CDT), warm detection threshold (WDT), thermal sensory limen (TSL), paradoxical heat sensation (PHS), cold pain threshold (CPT), heat pain threshold (HPT), mechanical detection threshold (MDT), mechanical pain threshold (MPT), MPS, DMA, wind-up ratio (WUR), vibration detection threshold (VDT), and PPT.

A thermal sensory testing device (THERMOCEPTION ANALYZER INTERCROSS-210; Intercross Inc., Tokyo, Japan) was used to perform thermal tests. A probe with a 25-mm^2^ surface area was used for all tests^13,20,21^. CDT and WDT were first measured using cold and warm stimuli, followed by the TSL. In the TSL, when the ramped stimulus reached a point where the participant first perceived the temperature as warm, the participant pressed a button. Subsequently, the direction of the temperature ramp was reversed and the thermode cooled down until the participant perceived a temperature change and again pressed the button. During this procedure, the number of occurrences of PHS was recorded, after which the CPT and HPT were determined^19^. Ramped stimuli of 1 °C/s were used with the procedure ending when the participant pressed the button^19,20^, and the participant was unable to see the computer screen during these measurements. The starting temperature on the right masseter muscle and right FDI was 32 °C, and cut-off temperatures were set at 0 °C for CPT and 50 °C for HPT^19,20^. Interstimulus interval between each thermal measurement was 4–6 s. CDT, WDT, CPT, and HPT were calculated as the mean of three measurements. Each measurement was repeated if the thermode slipped and provoked a mechanically induced pain sensation^19,20^.

MDT was measured using a standardized set of modified von Frey filaments (20 PIECE MONOFILAMENT KIT PRODUCT # 10-2000; Texas Medical Design , Texas, USA)^13,20,21^. The set of von Frey filaments contains monofilaments that exert different forces on bending. Each monofilament doubled the force exerted by the previous monofilament, ranging from 0.25 to 512 mN. All monofilaments were applied perpendicular to the examination site, with contact times ranging from 1 to 2 s. The five threshold measurements were made by applying a series of ascending and descending stimulus intensities, and the threshold value was calculated using the geometric mean of these five measurements^19,20^. Geometric mean was calculated as the average can be influenced by a few unrepresentative high judgements^21^.

For MPT measurements on the right masseter muscle and right FDI, a custom-made set of seven weighted pinprick stimulators was used^13,20,21^. The pinprick stimulators had a flat contact surface 0.2 mm in diameter. The range of forces of pinprick stimulators was from 8 to 512 mN, and contact time for the measurement areas was approximately 2 s. All pin-prick tests were made with the stimulator in a vertical position and perpendicular to the measurement area. The method-of-limits technique, similar to the one used to determine the MDT, was also used to determine the MPT. Similar to the MPT evaluation, seven weighted pinprick stimulators were used for MPS determinations.

DMA was estimated using three tactile stimulators including a cotton wisp, a cotton wool tip (Q-tip) attached to a flexible handle, and a disposable toothbrush (G.U.M #211 M; Sunstar Inc, Osaka, Japan). For the measurement of DMA, the three tactile stimulators were applied in a single stroke over a distance of 1–2 cm of the right masseter and FDI. MPS and DMA measurements comprised five stimulations with each of the 10 stimulators (7 weighted pinprick stimulators, 3 tactile stimulators) in randomized order according to the DFNS protocol^13,19^. In each of the total of 50 stimuli, the participant rated pain on a 0–100 numeric rating scale (NRS) with endpoints of 0 indicating no pain and 100 indicating most intense pain imaginable. MPS was calculated as the geometric mean of all numeric ratings using the seven weighted pinprick stimulators^19,20^. DMA value was calculated as the geometric mean of all numeric ratings using the three tactile stimulators^19,20^.

To measure WUR, 10 pinprick stimuli were repeated at a rate of 1 Hz according to a metronome and the perceived magnitude on the 0–100 NRS for pain was determined^19^. The WUR assessment used the same custom-made pinprick stimulators as used in MPT determinations. A pinprick stimulator that delivered a force that the participant perceived as slightly painful was selected, trying the 128-mN stimulator first. If the response from the participant to the 128-mN pinprick stimulus was 0 (not painful), WUR assessment was performed using a greater force. If the participant perceived the stimulus as intolerable, less force was used^13,19^. If a participant did not perceive the 512-mN stimulator as painful, the WUR assessment was abandoned. The participant was asked to give a pain rating representing the single stimulus, and the estimated mean over the whole series of 10 stimuli using a ‘0–100’ numerical rating scale. The whole procedure was repeated three times^12^.

VDT was assessed using a Rydel-Seiffer graded tuning fork (64 Hz, 8/8 scale)^14,19–21^. In the VDT assessment, the participant was asked to raise a hand to indicate when the vibration could no longer be sensed. A 9-point scale (0–8) was used to measure the intensity of vibration, with all values recorded to an accuracy of 0.5 units. The VDT assessment consisted of three trials, and the mean VDT from three trials was calculated for each participant.

PPT was measured using a digital pressure algometer (SOMEDIC ALGOMETER; Somedic Sales, Sösdala, Sweden) with a pinch handle and a probe surface area of 0.18 cm^2^. PPT assessment used a rate of increase in pressure of 50 kPa/s. The participant pressed a button to interrupt the stimulation when the first painful sensation was perceived. The PPT assessment consisted of three trials, using the mean value from three trials for analysis.

### Statistical analyses

Some QST parameters (with the exception of PHS and DMA) were not normally distributed, but normal distribution was achieved by logarithmic transformation (secondary normal distribution). Rolke et al. recommend executing log-transformation in the following QST parameters: CDT, WDT, TSL, MDT, MPT, MPS, ALL, WUR, and PPT^12,19^. All data are presented as the mean ± the standard deviations of the mean (SD). The normal distribution of variables was analyzed using the Shapiro–Wilk test (P < 0.05).

In the first experiment, a t-test was applied for comparisons of QST data between the two groups. Values of P < 0.05 were considered statistically significant. A z-score > 1.96 was regarded as a gain in somatosensory function, while a z-score  < − 1.96 was regarded as indicating a loss of somatosensory function^19,20,22^. Z-scores were calculated (subtracting the non-MPP group value mean from the MPP group value mean and dividing by the sample baseline SD) for all QST parameters. Z-score values > 0 indicate higher somatosensory sensitivity than the sample mean and values < 0 indicates lower sensitivity.

In the additional experiment, QST data were analyzed using two-way analysis of variance (ANOVA) with groups (MPP and non-MPP group) and time (pre- and post-application) as factors. When appropriate, ANOVA was followed by post-hoc Tukey testing to compensate for multiple comparisons. Data were analyzed using the SPSS statistical package (version 23.0; IBM Japan, Tokyo, Japan).

## Results

### Main experiment

There were no participants who reported any referred pain with 1.0-kg mechanical pressure stimulation for 2 s to the center of the right masseter muscle.

Table 1 shows the comparison of QST results between the MPP and non-MPP groups for the masseter muscle. MPS on the masseter muscle was significantly higher in the MPP group than in the non-MPP group (P < 0.05), and PPT was significantly lower in the MPP group than in the non-MPP group (P < 0.05) (Table 1).

**Table 1:** Comparison of quantitative sensory testing (QST) results between masseter muscle pain prone (MPP) group and non-masseter muscle pain prone (non-MPP) group for masseter muscle.

Applications	CDT (°C)	WDT (°C)	TSL (°C)	PHS (/3)	CPT (°C)	HPT (°C)	MDT (mN)	MPT (mN)	MPS (NRS)	DMA (NRS)	WUR (ratio)	VDT (/8)	PPT (kPa)
**MPP**	27.3	38.5	11.1	0.0	11.8	44.0	0.1	76.1	1.0	0.0	3.9	7.6	130.6
SD	(2.2)	(2.4)	(4.2)	(0.0)	(7.2)	(1.8)	(0.0)	(29.9)	(0.4)	(0.0)	(3.1)	(0.2)	(27.6)
**Non-MPP**	25.5	39.5	13.0	0.0	12.3	44.1	0.1	75.5	0.7	0.0	3.7	7.7	186.3
SD	(3.3)	(4.8)	(4.4)	(0.0)	(7.4)	(2.2)	(0.1)	(27.7)	(0.3)	(0.0)	(3.2)	(0.1)	(23.6)
P value	0.06	0.67	0.12	-	0.81	0.93	0.12	0.96	0.04*	-	0.51	0.24	0.01*

All data are presented as mean and standard deviations of the mean.

CDT = cold detection threshold (°C) ; WDT = warm detection threshold (°C) ; TSL = thermal sensory limen (°C) ; PHS = paradoxical heat sensation (score/3) ; CPT = cold pain threshold (°C) ; HPT = heat pain threshold (°C) ; MPT = mechanical pain threshold (mN) ; MPS = mechanical pain sensitivity (mean pain rating, 0–100) ; DMA = dynamic mechanical allodynia (NRS) ; WUR = wind-up ratio; MDT = mechanical detection threshold (mN) ; VDT = vibration detection threshold (score/8) ; PPT = pressure pain threshold (kPa) . (*P < 0.05, T-test).

**Figure 2:**
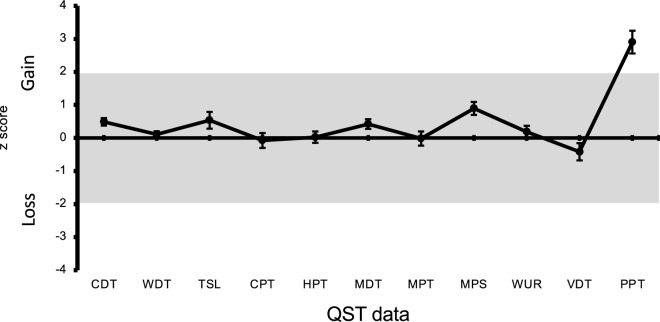
Z-scores on the Masseter for the MPP group. Error bars indicate the standard deviation of the mean. CDT = cold detection threshold ; WDT = warm detection threshold ; TSL = thermal sensory limen ; CPT = cold pain threshold ; HPT = heat pain threshold ; MPT = mechanical pain threshold ; MPS = mechanical pain sensitivity ; DMA = dynamic mechanical allodynia ; WUR = wind-up ratio; MDT = mechanical detection threshold ; PPT = pressure pain threshold .The gray zone (z score between − 1.96 and 1.96) represents the 95% confidence interval of baseline values.

Figure 2 shows z-scores on the masseter muscle for the MPP group based on the non-MPP data as reference values. Only the PPT values were outside the range between − 1.96 and 1.96.

Table 2 shows the comparison of QST results between the MPP and non-MPP groups for the FDI muscle with no significant differences for any QST parameter.

**Table 2:** Comparison of quantitative sensory testing (QST) results between masseter muscle pain prone (MPP) group and non-masseter muscle pain prone (non-MPP) group for first dorsal interosseous (FDI) muscle.

Applications	CDT (°C)	WDT (°C)	TSL (°C)	PHS (/3)	CPT (°C)	HPT (°C)	MDT (mN)	MPT (mN)	MPS (NRS)	DMA (NRS)	WUR (ratio)	VDT (/8)	PPT (kPa)
**MPP**	26.0	36.6	7.4	0.0	14.1	43.1	0.2	128.1	0.6	0.0	4.1	7.6	240.4
SD	(4.2)	(1.9)	(3.0)	(0.0)	(5.9)	(1.9)	(0.6)	(76.8)	(0.2)	(0.0)	(3.2)	(0.1)	(55.0)
**Non-MPP**	25.4	34.6	8.2	0.0	13.6	42.6	0.1	117.9	0.5	0.0	3.0	7.7	256.3
SD	(4.3)	(4.7)	(3.5)	(0.0)	(6.9)	(1.9)	(0.1)	(38.3)	(0.2)	(0.0)	(1.9)	(0.1)	(42.5)
P value	0.67	0.09	0.44	–	0.39	0.88	0.89	0.14	0.18	–	0.24	0.21	0.24

All data are presented as mean and standard deviations of the mean.

CDT = cold detection threshold (°C) ; WDT = warm detection threshold (°C) ; TSL = thermal sensory limen (°C) ; PHS = paradoxical heat sensation (score/3) ; CPT = cold pain threshold (°C) ; HPT = heat pain threshold (°C) ; MPT = mechanical pain threshold (mN) ; MPS = mechanical pain sensitivity (mean pain rating, 0–100) ; DMA = dynamic mechanical allodynia (NRS) ; WUR = wind-up ratio; MDT = mechanical detection threshold (mN) ; VDT = vibration detection threshold (score/8) ; PPT = pressure pain threshold (kPa) .

### Additional experiment

**Figure 3:**
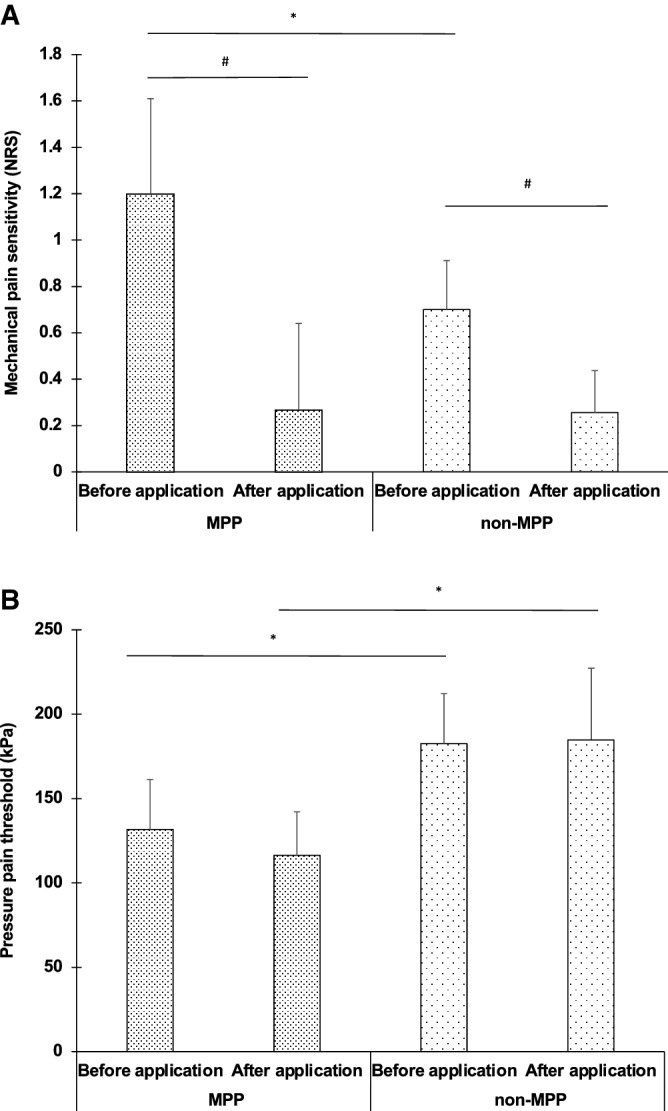
Comparison of MPS on Masseter muscle between before lidocaine application than after lidocaine application in the MPP and non-MPP groups (**A**), Comparison of PPT on Masseter muscle between before lidocaine application than after lidocaine application in the MPP and non-MPP groups (**B**). MPS on the masseter muscle was significantly higher in the MPP group than in the non-MPP group (*P < 0.05, Tukey post hoc test). In both the MPP and non-MPP groups, the MPS on the masseter muscle was significantly higher before lidocaine application than after lidocaine application (^#^P < 0.05, Tukey post hoc test). PPT was significantly lower in the MPP group than in the non-MPP group (*P < 0.05, Tukey post hoc test).

Table 3 shows the comparison of MPS, DMA, and PPT on the masseter muscle between before and after lidocaine application in the MPP and non-MPP groups. In both the MPP and non-MPP groups, the MPS on the masseter muscle was significantly higher before lidocaine application than after lidocaine application (P < 0.05) (Fig. 3A). After lidocaine application, no significant differences in MPS on the masseter muscle were evident in the MPP and non-MPP groups (Fig. 3A). In both the MPP and non-MPP groups, no significant differences in PPT on the masseter muscle were evident between before lidocaine application and after lidocaine application (Fig. 3B). Furthermore, both before lidocaine application and after lidocaine application, PPT on the masseter muscle was significantly lower in the MPP group than in the non-MPP group (P < 0.05) (Fig. 3B).

**Table 3:** Comparison of mechanical pain sensitivity (MPS), dynamic mechanical allodynia (DMA), and pressure pain threshold (PPT) on masseter muscle between before and after lidocaine application in the masseter muscle pain prone (MPP) and non-masseter muscle pain prone (non-MPP) groups.

	Applications	Before application		After application	
		Mean	SD	Mean	SD
**MPP**					
MPS	(NRS)	1.2	(0.4)	0.3	(0.4)
DMA	(NRS)	0.0	(0.0)	0.0	(0.0)
PPT	(kPa)	131.7	(29.6)	116.4	(25.8)
**Non-MPP**					
MPS	(NRS)	0.7	(0.2)	0.3	(0.9)
DMA	(NRS)	0.0	(0.0)	0.0	(0.0)
PPT	(kPa)	182.6	(29.0)	184.7	(42.4)

All data are presented as mean and standard deviations of the mean. MPS = mechanical pain sensitivity (mean pain rating, 0–100); DMA = dynamic mechanical allodynia (NRS); PPT = pressure pain threshold (kPa).

## Discussion

The present study investigated whether short-lasting pressure-evoked masseter muscle pain is associated with alterations in somatosensory sensitivity of the overlying skin in healthy individuals. The main findings in this study were: (1) MPS on the masseter muscle (homotopic) was significantly higher in the MPP group than in the non-MPP group; and (2) no significant differences in MPS before and after lidocaine patch application were evident between MPP and non-MPP groups. As expected the PPTs were lower in the MPP group compared to non-MPP group. There were no impact on thermal or tactile sensitivity.

According to the main experiment, MPS on the masseter muscle was significantly higher and PPT was significantly lower in the MPP group than in the non-MPP group. The present study found no significant differences in CDT, WDT, TSL, PHS, CPT, HPT, MDT, MPT, DMA, WUR or VDT on masseter muscle between the MPP and non-MPP groups in the main experiment. MPS was assessed using the same set of seven weighted pin-prick stimuli to obtain a stimulus–response function for pinprick-evoked pain, designed to detect pin-prick hyperalgesia^22^. Meints et al. found that patients with chronic low back pain demonstrated greater deep-tissue hyperalgesia as well as increased sensitivity for mechanical punctate pain compared to pain-free controls^23^. In addition, it is well known that widespread hyperalgesia on quadriceps femoris muscle in deep tissue is a common finding in patients with muscle pain and could be related to a dysfunction of the descending inhibitory system^24^. Puta et al. have reported that widespread changes of somatosensory sensitivity were found in chronic low back pain patients. Furthermore, significantly enhanced pain thresholds were found not only at the back, but also at a non-painful hand^25^. While the innervating nerves and anatomical location of chronic pain area of previous studies differ from those involved in masseter muscle pain, increased pain sensitivity may occur in the skin overlying the masseter muscle. The present findings suggest that masseter muscle pain is at least partially related to subjective changes of the mechanical pain sensitivity of the skin overlying the masseter muscle.

Costa et al. investigated the effect of experimental short-lasting muscle pain on the tactile sensitivity of the skin overlying the masseter muscle^11^. Glutamate-evoked jaw muscle pain is well known to simulate aspects of myogenous temporomandibular disorders^26^. Costa et al. found that the MDT on the masseter muscle was significantly lower before glutamate injection than after glutamate injection and concluded that experimental short-lasting muscle pain impair touch perception^11^. That result appears to conflict with the results from the present study, potentially due to several factors. However, Svensson et al. previously demonstrated mechanical hyperesthesia to pin prick stimuli following prolonged nociceptive stimulation of the masseter muscle^27^. To further clarify the mechanism of normal physiological masseter muscle pain, studies will need to investigate the effect of different types of masseter pain, e.g., post-exercise muscle soreness or nerve-growth factor-induced sensitization on somatosensory sensitivity.

According to the additional experiment, the MPS on the masseter muscle was significantly lower after lidocaine application than before lidocaine application in both the MPP and non-MPP groups. On the other hand, no difference in MPS on the masseter muscle was seen between MPP and non-MPP group after lidocaine patch application. Wehrfritz et al. reported that lidocaine tape applied to healthy skin on the volar forearm can alter the mechanical pain threshold, mechanical wind-up, and tactile threshold^28^. Okayasu et al. also reported NRS pain intensity of the cheek skin decreased after application of 8% lidocaine spray^29^. In addition, Pillai et al. found that 5% local anesthetic agent containing 2.5% lidocaine and prilocaine application caused significant somatosensory loss in thermal and mechanical parameters CDT, WDT, TSL, CPT, MDT, MPT, MPS, and VDT when compared to baseline in the right infraorbital (V2) region^30^. Inada et al. investigated the efficacy of lidocaine tape for alleviating the pain associated with a stellate ganglion block^18^. They also found that the lidocaine tape reduced visual analog scale evaluations of pain after application for as little as 7 min^18^. The results for MPS from the main and additional experiments suggest subjective change of mechanical pain within the range of effect of the topical lidocaine. Further studies are needed to investigate the subjective change of mechanical pain sensitivity for the skin over the masseter muscle, to elucidate the mechanisms of related pain among patients with masseter muscle/fascial pain. For PPTs, no differences on the masseter muscle were seen between before and after lidocaine patch application in the MPP and non-MPP groups. However, a significant difference was evident between the MPP and non-MPP groups. Past studies have demonstrated no difference in PPT sensitivity after lidocaine patch application^11,31^. Such results agree well with past results^11,31^. The lack of difference in PPT on the masseter muscle between before and after lidocaine patch application in the MPP and non-MPP groups may indicate that pressure pain sensation in the human masseter muscle was not derived predominantly from cutaneous tissues, but rather from the muscle itself. No difference in DMA on the masseter muscle was evident between before and after lidocaine patch application in the MPP and non-MPP groups. This finding was not unexpected, given that only healthy participants were recruited to this study.

It must be acknowledged that even though the present study applied mechanical devices to standardize palpation for participants and allow division into two groups, then the evoked pain was in any case short-lasting (seconds). It may therefore not be an effect of ongoing nociceptive input which alters the MPS in MPP individuals but rather a trait. Not surprisingly, the PPTs were also lower in the MPP but no other of the standardized QST measures indicated any significant difference. It could be of interest to test if participants who report referred pain sensations in response to longer and more intense palpation pressure would display any difference in homotopic and heterotopic (referred pain area) somatosensory sensitivity compared to participant who only report local pain on palpation. Therefore, further research into levels of pressure-evoked pain, including the duration of pain, need to be conducted. In addition, psychological factors were not investigated in the present study, as only healthy participants were recruited. However, pain perception is well known to occur with a high frequency of psychological comorbidities and sleep deprivation^31,32^. Future studies will thus be required to standardize other participant conditions.

## Conclusion

Brief, acute pain in the masseter muscle is linked to increased MPS which can be reversed by transient deafferentation of the superficial nociceptive input. Chronic pain in the masseter muscle could therefore influence homotopic sensitivity.
